# Respiratory Morbidity Following Foreign Body Aspiration Among South Indian Children: A Descriptive Study

**DOI:** 10.7759/cureus.3629

**Published:** 2018-11-23

**Authors:** Narayanan Parameswaran, Sarthak Das, Niranjan Biswal

**Affiliations:** 1 Paediatrics, Jawaharlal Institute of Postgraduate Medical Education and Research, Puducherry, IND

**Keywords:** children, foreign body aspiration, respiratory morbidity, south india

## Abstract

Aim

Our work aimed to study the clinical features and radiological signs of foreign body aspiration in South Indian children.

Materials and methods

We conducted this prospective cross-sectional study for one year in the Department of Paediatrics, Jawaharlal Institute of Postgraduate Medical Education & Research (JIPMER), Pondicherry, India in 56 cases. Our study included children younger than 14 years attending our hospital with a history of foreign body aspiration with or without respiratory distress, suspected cases of foreign body aspiration showing evidence of unilateral hyperinflation with or without evidence of collapse of the opposite lungs on chest X-ray (posterior-anterior view), and unexplained cough associated with X-ray evidence of unilateral hyperinflation or bronchiectasis. Emergency and elective rigid bronchoscopy were performed as per the clinical scenario, and patients were discharged from the hospital after stabilization. They were asked to return for two-week, three-month, and six-month follow-up evaluations.

Results

The most common age group of patients with foreign body aspiration was six to 18 months old. A definite history of foreign body aspiration was obtained in only 32 patients (57%). Most patients (n = 22; 68.75%) presented to the hospital within one to seven days of the foreign body aspiration. Respiratory distress was the most common clinical feature, present in 96% of our patients. The most common radiographic feature in our study was obstructive emphysema, seen in 33 patients (58.9%). Foreign bodies were retrieved from 40 patients (71.4%), and no foreign body could be found via bronchoscopy in 16 patients. The most common foreign body was groundnut (n = 27; 67.5%). Only four patients had inorganic foreign bodies. The most common site of aspirated foreign bodies was the right main bronchus (n = 14; 35%). Only two patients (3.57%) had persistent pneumonia that cleared up radiologically after three months and was culture negative for bacteria.

Conclusions

Strong clinical suspicion of foreign body aspiration based on history and early bronchoscopy can reduce the morbidity and mortality due to foreign body aspiration in children.

## Introduction

Foreign body aspiration represents a life-threatening clinical problem in pediatric emergency departments and very often leads to fatal acute respiratory failure and death, especially in preschool-aged children [1,2]. Foreign body aspiration into the human tracheobronchial tree results in a wide spectrum of presentations ranging from asymptomatic to death [3]. The wide variety of clinical presentations and outcomes depends on factors such as the age of the child, the caliber of the airway, the location and extent of airway obstruction, and, especially, the availability of a health care expert in developing countries [1,4].

The most life-threatening consequence in foreign body aspiration is acute respiratory distress, which is, fortunately, not common. Delayed/non-removal of foreign bodies can lead to a spectrum of clinical features from simple coughing and wheezing to recurrent or nonresolving respiratory sequelae like chronic pulmonary infections, bronchiectasis, and lung abscess in later life. These presentations sometimes mimic other respiratory illness like asthma, pneumonia or tracheobronchitis, and, unfortunately, they are wrongly managed by pediatricians. The diagnosis of a tracheobronchial foreign body requires a high index of suspicion and early management by the pediatricians to prevent morbidity and mortality due to delayed or inappropriate diagnosis [3,4].

This study aims to examine respiratory morbidity following foreign body aspiration in children including clinical symptoms, signs, complications, and risk factors.

## Materials and methods

We conducted this prospective study for one year in the Department of Paediatrics, Jawaharlal Institute of Postgraduate Medical Education & Research (JIPMER), in Pondicherry, India. All the children included in the study were younger than 14 years, attended our hospital with a history of foreign body aspiration with or without respiratory distress, had suspected cases of foreign body aspiration showing evidence of unilateral hyperinflation with or without evidence of collapse of the opposite lungs in X-ray chest (posterior-anterior view), and unexplained cough associated with X-ray evidence of unilateral hyperinflation or bronchiectasis.

Those patients with repeated choking, stridor, severe respiratory distress, cyanosis or other emergency condition received rigid bronchoscopy when necessary. Other patients who were stable received supportive measures (e.g., antibiotics and bronchodilators) and were clinically investigated (via hemoglobin, total lung capacity, differential count, and chest X-ray). Patients underwent elective bronchoscopy as soon as possible. We carefully observed these patients in the ward for any evidence of increased respiratory distress that can occur with the migration of the foreign body.

Rigid bronchoscopy was done under general anesthesia for all the patients. The bronchoscopy was performed by a skilled otolaryngology consultant with vast experience in paediatric bronchoscopy assisted by senior residents using a Karl Storz rigid bronchoscope of appropriate size. Following bronchoscopy, we observed all patients in the paediatric intensive care unit for the development of any complications. Chest radiographs were taken six hours after the procedure. The patients were discharged from the hospital once they were stable and had no signs of respiratory distress. They were asked to come for follow-up at regular intervals of two weeks, three months and six months.

Thorough clinical assessments were conducted at follow-up evaluations, and repeat chest radiographs were taken to look for any residual long-term complications (i.e., bronchiectasis, collapse or emphysema). The follow-up period was up to six months.

Statistical analysis was done using Chi-square test and Fisher exact test.

## Results

Fifty-six patients satisfied the inclusion criteria for our study. Of the 56 patients, 40 (71.4%) were boys and 16 (28.6%) were girls.

The most common age group in which foreign body aspiration was seen in our study was the six- to 18-month age group comprising 34 (60.7%) patients. Only nine patients (16%) were older than two years. The youngest patient was seven months old, and the oldest was 10 years.

A definite history of foreign body aspiration could be obtained in only 32 patients (57%). Of the 24 patients who with no history of foreign body aspiration, foreign bodies were removed from 13 (54.1%) cases.

Among 32 patients who gave a history of foreign body aspiration, only four patients (12.5%) were brought to the hospital within 24 hours of aspiration. Most cases (n = 22; 68.75%) presented to the hospital one to seven days following the foreign body aspiration, five patients (15.62%) presented one week to one month after aspiration, and one patient (3.12%) presented after one month following aspiration. The earliest time a patient reached the hospital was five hours, while the longest time taken was six months.

Respiratory distress was the most common clinical feature, present in 96% of our patients, followed by cough (87.5%), evidence of decreased air entry (80%), and mediastinal shift (44.6%) on examination. Fever was present in 62.5% of our children. Fever was significantly more common in children who presented more than 24 hours after the aspiration (P = 0.04). However, the difference in fever between the organic and inorganic foreign body groups was not statistically significant (P > 0.05) in our study (Table 1).

**Table 1:** Clinical features of children with suspected foreign body aspiration. P = 0.04 for decreased air entry. Others not significant.

CLINICAL FEATURES	POSITIVE BRONCHOSCOPY		NEGATIVE BRONCHOSCOPY		P VALUE
	NUMBER	PERCENTAGE (%)	NUMBER	PERCENTAGE (%)	
History of aspiration	25	62.5	7	43.8	0.55
Cough	35	87.5	14	87.5	1.29
Respiratory distress	39	97.5	15	93.8	0.61
Stridor	3	7.5	1	6.3	0.40
Wheeze	11	27.5	3	18.8	0.08
Fever	16	40	7	43.8	2
Recurrent pneumonia	4	10	1	6.3	1.02
Intermittent dyspnoea	6	15	2	12.5	0.83
Purulent expectation	2	5	0	0	0.57
Hoarseness of voice	7	17.5	1	6.3	0.90
Expulsion of inhaled bits	2	5	3	18.8	1.02
Subcutaneous emphysema	2	5	0	0	0.07
Mediastinal shift	18	45	7	43.8	1
Chest deformities	4	10	2	12.5	0.68
Decreased air entry	35	87.5	10	62.5	0.04

Five cases gave the history of an expulsion of inhaled bits, and two had subcutaneous emphysema on examination.

All the clinical features were compared between the foreign body positive and negative bronchoscopy groups to determine any significant difference between the two groups (Table 1). Only decreased air entry to the lung with the foreign body was found to be significantly more common in the positive bronchoscopy group compared to the negative bronchoscopy group (P = 0.04).

The most common radiographic feature in our study was obstructive emphysema, which was seen in 58.9% of patients (n = 33; unilateral, 48.2%; bilateral 10.7%). Infiltrate was found in 25% (n = 14), collapse was found in 23.2% (n = 13), and compensatory emphysema was seen in 10.7% of patients (n = 6). Radiographs were unremarkable in 3.5% of cases. The average duration of presentation after aspiration was 3.59 days for those with obstructive emphysema and 40 days for patients with collapse.

The sensitivity of chest radiograph in diagnosing foreign body aspiration was 95.2% with a positive predictive value of 78.4%. However, chest radiography had a specificity of only 21.4% (Table 2).

**Table 2:** Diagnostic accuracy of chest radiograph in foreign body aspiration. Total number of patient (n) = 56.

CHEST RADIOGRAPHY	BRONCHOSCOPY FINDINGS	
	Foreign body present	Foreign body absent
Suggestive of foreign body aspiration	40 (71.42%)	11 (19.64%)
Normal	2 (03.57%)	3 (05.35%)

Fifty-seven bronchoscopies were performed in 56 patients; bronchoscopy had to be repeated in one patient due to a failed first attempt. Of the 56 patients who underwent bronchoscopy, foreign bodies were retrieved from 40 (71.4%), and no foreign body was found in 16 patients. Among the 16 patients who had negative bronchoscopy results, eight (50%) had purulent secretions, three (18.7%) had edema, two (12.5%) had mucosal congestion, one (6.25%) had granulations, and five (31.25%) had no specific findings. The most common foreign body was groundnut (n = 27; 67.5%; Table 3).

**Table 3:** Types of foreign bodies retrieved by bronchoscopy.

TYPE OF FOREIGN BODY	NUMBER	PERCENTAGE
Ground nut	27	67.5
Dhal piece	2	5
Pepper	1	2.5
Tamarind seed	2	5
Coconut piece	1	2.5
Mango piece	1	2.5
Custard apple seed	1	2.5
Unknown seed	1	2.5
Charcoal piece	1	2.5
Plastic whistle	1	2.5
Rubber piece	1	2.5

The most common site of aspirated foreign bodies was the right main bronchus (n = 14; 35%) followed by the left main bronchus (n = 12; 30%). The foreign body was located in the trachea in six cases (15%), and one patient (2.5%) had a subglottic foreign body. Foreign bodies at multiple locations were seen in three cases (7.5%).

Thirty-two patients came for follow-up evaluation after their bronchoscopy. Only two of them had persistent pneumonia that cleared up in three months and was culture negative for bacteria (Table 4).

**Table 4:** Follow-up of bronchoscopy patients.

FOLLOW UP DURATION	SYMPTOMATIC n (%)	PERSISTENT PNEUMONIA n (%)	COLLAPSE n (%)	BRONCHIECTASIS n (%)	ASYMPTOMATIC n (%)	TOTAL (n)
Two weeks	1 (4)	2 (8)	0	0	22 (88)	25
Three months	0	0	0	0	14 (100)	14
Six months	0	0	0	0	3 (100)	3

Tuberculosis was ruled out in these cases with suitable investigations. One patient who presented more than one month after the aspiration of groundnut was asymptomatic at follow-up. No other complications were noted in our study. The frequent complications following foreign body aspiration were persistent preoperative pneumonia, intraoperative bleeding from the site of foreign body lodging, and postoperative bronchospasm.

There were no deaths in our study.

## Discussion

The age group with the highest incidence of foreign body aspiration in our study (those aged six months to 18 months) aligned with the findings of studies done in India, the US, and Israel [3,5,6]. Children younger than two years have inadequate dentition and immature swallowing coordination. These children are most vulnerable to foreign body aspiration due to their tendency to put objects into their mouths as a means of exploration due to their oral orientation as toddlers [3]. Additionally, teeth cannot chew completely, and consequently, the food retained in the mouth for longer times increases the chance for aspiration of solid material [7].

Our male:female ratio for aspiration is 2.5, which is similar to that reported by Naragund et al. and McGuirt et al. [3,8]. This predominance among boys may be due to their active and adventurous nature.

Due to their ambulatory nature and the lack of supervision by the parents, children younger than two years are more prone to acute foreign body aspiration without witness [7]. Rothmann and Boeckman found a definitive history of foreign body aspiration 74.9% of cases in their study, which is much higher than what we observed in our patient population [5].

Kaur et al. and Naragund et al. [9,3] noted that most patients present within one to seven days of aspiration, as we also noted [9,3], yet a Turkish study reported most patients presented to the hospital within 24 hours of aspiration, and very few presented in the days and months after aspiration [7]. The delay in presentation in our study may be due to the delay in referral by the local practitioner who failed to suspect foreign body aspiration as a possible cause for the child’s problem. Early hospitalization in the Turkish study could be related to an increased awareness of foreign body aspiration among the significantly more literate population of developed countries in comparison to India.

While respiratory distress was the most common clinical feature in our study, Kaur et al., McGuirt et al., and Merchant et al. reported their most common presenting symptoms were cough, followed by respiratory distress, wheezing, and fever [8-10]. These discordant results could be related to the time gap between foreign body aspiration and admission. In other words, the aspiration was diagnosed earlier in more patients of the other studies before their conditions could progress to respiratory distress [7,8].

Our study had a higher incidence of fever than what was reported in other similar studies [8-10].

Evidence of decreased air entry in the hemithorax due to foreign body was more common in our study than in another study done in India [3]. Other reports noted that more than half of their children displayed a decrease in respiratory sounds on one side of the thorax or in one of the pulmonary lobes during auscultation [8-10].

The first diagnostic modality in the suspicion of foreign body aspiration is a simple chest X-ray due to easy availability of X-ray equipment at most health centres. Obstructive emphysema was common in our study as well as other studies [3,11]. Patients in our study with radiological evidence of bilateral emphysema had a foreign body lodged in the trachea or at the carinal level. None of our patients with bilateral emphysema had foreign bodies partially obstructing both the main bronchi.

A study in Brazil showed higher sensitivity and a lower specificity of radiography for diagnosing foreign bodies than our study [11]. This variation solely depends upon the nature of the foreign body.

Detection rate of opaque foreign body on chest X-ray was low in our study as well as another Indian study [3], but 20% to 25% of patients in studies in developed countries [11,12]. The disparity between the findings is likely due to differences in the geographic zone, cultural patterns, the types of foreign bodies, and the socioeconomic status of each country.

Foreign bodies were retrieved following bronchoscopy in 60 cases (87%) and 74 cases (71.8%) in Brazilian and Iranian studies, respectively, which is almost similar to our findings [11,13]. The negative bronchoscopy rate in our study was slightly higher compared to other studies.

Vegetative foreign body was the most commonly aspirated in our study along with several other studies [3,7,11,14]. Groundnut is cheap and is commonly offered to children by parents or siblings as a means to console them. Children younger than two years have abnormal neuromuscular coordination and no molar and premolar teeth. A lack of monitoring by the family members during feeding time makes these children more susceptible to foreign body aspiration [3]. Nuts expand by absorbing water and break into multiple pieces which can easily pass into distant airways, making the pieces inaccessible for removal during bronchoscopies.

Foreign bodies predominantly lodge in the right main bronchus (as noted in this and other studies [8-12]) due to its anatomical location and large diameter.

The reported long-term complications of foreign body aspiration can include bronchiectasis, recurrent pneumonitis, and pyopneumothorax [15]. We did not encounter these long-term complications in any of our patients. None of our patients had exercise intolerance on follow-up.

## Conclusions

Foreign body aspiration is a common life-threatening emergency in developing countries. Inadequate supervision and the exploratory nature of children below two years make them more vulnerable to foreign body aspiration. So especially in developing countries children less than two years of age should be monitored by parents or by the elder members of families during feeding very meticulously. Strong clinical suspicion of foreign body aspiration based on history and diminished air entry in a hemithorax and early and meticulous radiography of the chest immensely help in deciding in favor of bronchoscopy. Observation in an intensive care unit and early bronchoscopy can lead to a speedy recovery, prevents long-term complications and finally the mortality results from foreign body aspiration.
